# Mucosal-associated invariant T (MAIT) cells provide B-cell help in vaccinated and subsequently SIV-infected Rhesus Macaques

**DOI:** 10.1038/s41598-020-66964-0

**Published:** 2020-06-22

**Authors:** Mohammad Arif Rahman, Eun-Ju Ko, Farzana Bhuyan, Gospel Enyindah-Asonye, Ruth Hunegnaw, Sabrina Helmold Hait, Christopher James Hogge, David J. Venzon, Tanya Hoang, Marjorie Robert-Guroff

**Affiliations:** 1 Vaccine Branch, Center for Cancer Research, National Cancer Institute, National Institutes of Health, Bethesda, MD USA; 2 Laboratory of Clinical Immunology & Microbiology, National Institute of Allergy and Infectious Diseases, National Institutes of Health, Bethesda, MD USA; 3 Biostatistics and Data Management Section, National Cancer Institute, National Institutes of Health, Bethesda, MD 20892 USA; 4 Present Address: College of Veterinary Medicine and Interdisciplinary Graduate Program in Advanced Convergence Technology & Science, Jeju National University, Jeju, 63243 Korea

**Keywords:** Immunology, Innate immune cells, Innate lymphoid cells, Microbiology, Virology, Retrovirus

## Abstract

Mucosal-associated invariant T (MAIT) cells help combat opportunistic infections. Thus, MAIT cells are of interest in HIV/SIV vaccination and infection. We investigated MAIT cell dynamics and function in rhesus macaque blood and bronchoalveolar lavage (BAL) following mucosal adenovirus (Ad)-SIV recombinant priming, intramuscular SIV envelope boosting and infection following repeated low-dose intravaginal SIV exposures. Increased frequencies of blood MAIT cells over the course of vaccination were observed, which were maintained even 12-weeks post-SIV infection. BAL MAIT cells only increased after the first Ad immunization. Vaccination increased MAIT cell levels in blood and BAL expressing the antiviral cytokine IFN-γ and TNF-α and the proliferation marker Ki67. Upon T cell-specific α-CD3, α-CD28 stimulation, MAIT cells showed a greater capacity to secrete cytokines/chemokines associated with help for B cell activation, migration and regulation compared to CD3^+^MR1^−^ cells. Culture of MAIT cell supernatants with B cells led to greater tissue like memory B cell frequencies. MAIT cell frequencies in blood and BAL correlated with SIV-specific antibody levels in rectal secretions and with SIV-specific tissue resident memory B cells. Overall, SIV vaccination influenced MAIT cell frequency and functionality. The potential for MAIT cells to provide help to B cells was evident during both vaccination and infection.

## Introduction

Mucosal-associated invariant T (MAIT) cells provide a rapid, innate-like response against bacterial as well as viral infections. In bacterial infection, MR1, a highly conserved MHC-related antigen-presenting protein, presents small molecule ligands from the riboflavin biosynthesis pathway to MAIT cells and activates them to control infection^1^. Unlike bacterial pathogens, many viruses lack metabolic pathways to activate MAIT cells through MR1 and activate them instead in an IL-18-dependent manner, together with IL-12, IL-15, and other pro-inflammatory cytokines such as IFNα/β^2^. MAIT cells secrete cytokines, such as IL-17 A and IL-22^3,4^, which help maintain epithelial integrity and mucosal homeostasis, critical for controlling HIV-related opportunistic infections^5^. Furthermore, activated MAIT cells from human blood predominantly secrete the proinflammatory cytokines, IFN-γ and TNF-α^6^, important in containing viral infection. Therefore, their frequency and function in HIV-infected individuals are of considerable interest^7^. Although, MAIT cells are not preferentially infected by HIV^8^, an early depletion of MAIT cells from the peripheral circulation has been observed in HIV infection^9,10^, with significant depletion seen during the first year of disease progression^8,11^. Similarly, low frequencies of MAIT cells were observed in chronically SIV-infected rhesus macaques compared to uninfected animals^12^. In contrast, in the pigtail monkey SIV/SHIV infection model, no consistent impact of SIV/SHIV infection on peripheral MAIT cell frequency was observed during an 81-week post challenge period^13^. Although MAIT cells have been extensively studied in humans, few longitudinal studies have examined the dynamics of MAIT cells in SIV/SHIV infected macaques to resolve these differences.

MAIT cells play a significant role in the first line of defense against different pathogens in a non-antigen-specific manner, but they can be induced by various infections or vaccination. Mice intranasally challenged with sublethal doses of the live vaccine strain of *F. tularensis* recruited large numbers of MAIT cells into the lungs^14^. *Legionella longbeachae* infection of mice induced MR1-dependent MAIT cell activation and rapid pulmonary accumulation of MAIT cells associated with immune protection in immunocompetent host animals^15^. Human volunteers receiving an attenuated strain of *Shigella dysenteriae*-1 as a potential oral vaccine exhibited greater MAIT cell activation^16^. HIV^+^ patients therapeutically immunized with GTU-Multi HIV clade B DNA vaccine^17^ showed an increased frequency of MAIT cells^18^. In rhesus macaques, MAIT cell activation *in vivo* has been observed in response to both Bacillus Calmette-Guerin vaccination and *Mycobacterium tuberculosis* infection^19^. Thus, vaccination as well as some infections can cause activation and accumulation of MAIT cells. No report, however, has yet shown the effect of SIV vaccines on MAIT cell frequency and functionality.

T follicular helper (T_FH_) cells^20^ and other T cell subsets, such as invariant natural killer T (iNKT) cells^21^, γδ T cells^22^, and MAIT cells^23^, have been shown to provide help to B cells. In healthy human donors, *in vitro* assays demonstrated that activated MAIT cells secrete factors that act on B cells to promote differentiation of memory cells into plasmablasts (PB) and increase antibody production^23^. A positive correlation between MAIT cell frequency and *Vibrio cholerae* lipopolysaccharide‐specific IgA and IgG antibody responses^24^ has been reported. Moreover, vaccination with attenuated *S. dysenteriae* led to a lipopolysaccharide-specific antibody-secreting cell response associated with activated MAIT cells^16^, further suggesting that MAIT cells might act as B helper cells. This possibility has not been investigated in SIV vaccinated or infected rhesus macaques.

Here we conducted a longitudinal study in rhesus macaques with two specific aims. The first was to elucidate the dynamics and functionality of MAIT cells in blood and at a mucosal site over the course of a SIV vaccine regimen and following subsequent SIV infection. We found that changes in MAIT cell responses, including frequency and cytokine production, were largely due to vaccination with a replicating Adenovirus (Ad) vector and alum adjuvant rather than the SIV immunogens. We observed that vaccination increased MAIT cell frequency and functionality in blood; however, the effect of vaccination was not as evident in bronchoalveolar lavage (BAL) cells, investigated as the vaccine regimen targeted mucosal sites including the upper respiratory tract. Unlike HIV infection, in the early phase of SIV disease progression at 12 weeks post-infection (wpi), no significant decrease of MAIT cell frequency in blood and BAL compared to pre-infection levels was observed. Secondly, as viral-specific antibody responses have been shown to be important for HIV vaccine efficacy^25–27^ we investigated whether MAIT cells over the course of vaccination possess the ability to help B cells. We observed that MAIT cells secrete cytokines that might help mediate the class switching, migration and activation of B cells. Upon vaccination, the frequency of MAIT cells in blood and BAL correlated with mucosal SIV-specific memory B cells and with antibody levels at a later time point, suggesting MAIT cells influence tissue resident memory B cell frequency as well as SIV-specific antibody production. Overall the Ad-based vaccine regimen modulated MAIT cell responses, which in turn enhanced B cell functionality.

## Results

### MAIT cell dynamics upon vaccination and subsequent SIV infection

**Figure 1:**
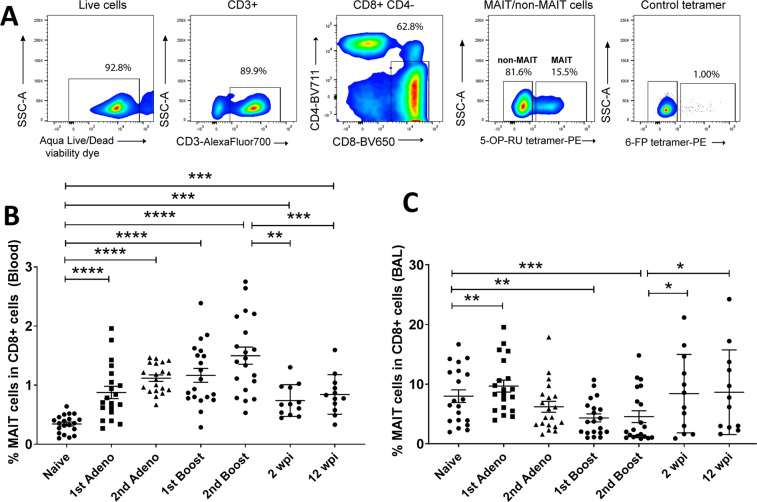
Quantitation of MAIT cell frequency in blood and BAL fluid upon vaccination and subsequent SIV infection in rhesus macaques. (**A**) Representative gating strategy of MAIT cells. (**B**,**C**) Frequency of MAIT cells in blood and BAL over the course of vaccination and infection. Data of (**B,C**) were analyzed by the Wilcoxon paired test. Horizontal and vertical bars denote mean and SEM. *p < 0.05, **p < 0.01, ***p < 0.001, ****p < 0.0001.

We studied MAIT cells in blood and in BAL fluid over the course of vaccination and SIV infection (described in Materials and Methods) in rhesus macaques. We defined MAIT cells as CD3^+^CD4^−^CD8^+^ cells binding to 5-OP-RU MR1 tetramers (Fig. 1A)^19^, focusing on the CD8^+^ MAIT cell subgroup. Based on expression of CD4 and CD8, MAIT cells are divided into different subgroups. In healthy humans, CD8^+^ and DN (CD8^−^CD4^−^) MAIT cells are the predominant populations in blood, whereas CD4^+^ and DP (CD8^+^CD4^+^) cells are present less frequently^28,29^. In mice the majority of MAIT cells are DN cells^30^. Here, using blood and BAL samples from 20 naïve macaques, we determined the frequencies of the various MAIT cell subgroups (gating strategy shown in Supplemental Fig. S1). The mean percentages of CD8^+^CD4^−^, DP, CD8^−^CD4^+^ and DN cell populations in live CD3^+^MR1^+^ cells were 36.3%, 2.9%, 15.8% and 44.9% in blood and 66.8%, 5.86%, 8.11% and 19.2% in BAL of the naïve macaques. Thus, as in humans, CD8^+^ and DN MR1^+^ cells are predominant in rhesus macaques. As gene analysis data has shown that CD8^+^ MAIT cells in humans have superior functionality following stimulation compared to DN cells^31^, here we focused on the CD8^+^ subgroup.

We initially observed that changes in MAIT cell frequencies and expression of cytokines were similar between the vaccinated and control macaques (Supplemental Figs. S2–S4), reflecting innate immune responses to the replicating adenovirus type 5 host range mutant (Ad5hr) vector and adjuvant administered to both groups. Therefore, data in both groups have been combined. Compared to pre-immunization (naïve) levels, a higher frequency of MAIT cells was observed in blood upon vaccination and subsequent infection (Fig. 1B). In acute (2 wpi) and chronic (12 wpi) SIV infection, the frequency of MAIT cells decreased compared to that observed in vaccinated macaques but remained elevated compared to the naïve level. To determine MAIT cell frequencies at a mucosal site we investigated BAL samples. As the vaccine regimen was designed for subsequent intravaginal SIV challenges, we did not want to disrupt that site by taking vaginal biopsies. Additionally, rectal biopsy tissues were used in other cellular and humoral immune assays and were not readily available. However, since, mucosal Ad5hr priming immunizations were administered to the upper respiratory tract, evaluation of BAL samples was relevant. An increase in MAIT cell frequency in BAL samples was observed after the 1^st^ adeno vaccination, however, it returned to the pre-immunization level after the 2^nd^ vaccination and remained unchanged throughout the course of vaccination. After infection, an increased frequency of MAIT cells was observed compared to levels following the last vaccination but was similar to levels in naïve cells (Fig. 1C). The decreased level of MAIT cells in BAL following the initial response to the 1^st^ adeno immunization might be due to migration of the cells from lung to other organs, however, further work will be needed to determine this. Overall, vaccination increased the frequency of MAIT cells in the blood of rhesus macaques over pre-immunization levels, and this increase was maintained following infection.

### Comparison of cytokine expressing MAIT cells and CD8^+^ non-MAIT cells in the blood and BAL upon vaccination and subsequent SIV infection

**Figure 2:**
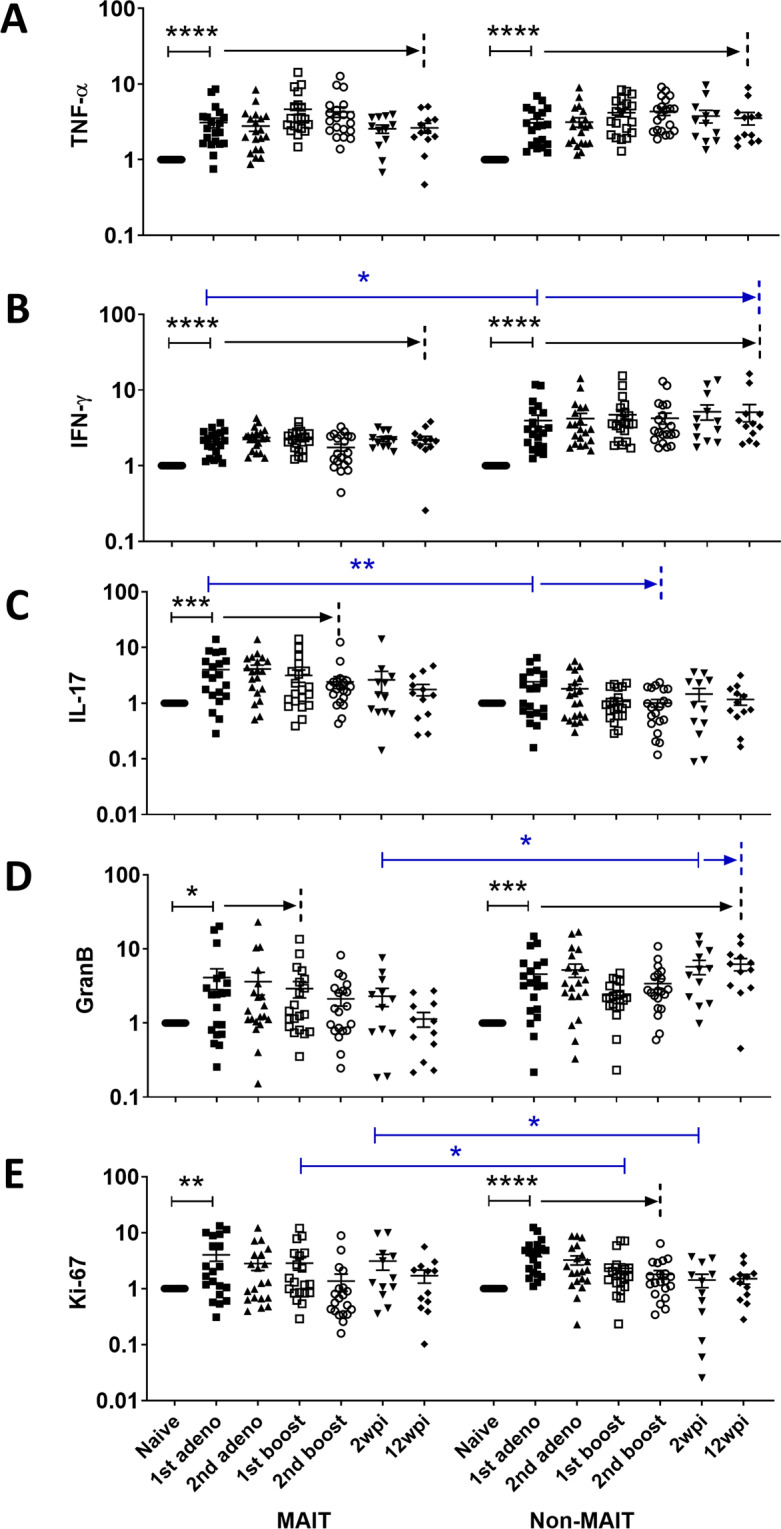
Comparison of fold change of cytokine expressing MAIT and CD8^+^ non-MAIT cell subsets in blood. Fold change following PMA/Ionomycin stimulation of (**A**) TNF-α^+^, (**B**) IFN-γ^+^, (**C**) IL-17^+^, (**D**) Granzyme B^+^ and (**E**) Ki-67-expressing MAIT and CD8^+^ non-MAIT cells compared to pre-samples (naïve) over the course of vaccination and infection. Significant differences between cytokine-expressing MAIT and non-MAIT cells at indicated time points are also shown. Data of (**A**–**E**) were analyzed by the Wilcoxon paired test. Horizontal and vertical bars denote mean and SEM. *p < 0.05, **p < 0.01, ***p < 0.001, ****p < 0.0001. The black arrows indicate persistence of significant differences compared to the naïve sample up to the time point marked by the dashed line. For simplicity, p values for each comparison ranging from <0.05 to <0.0001 are not shown. Similarly, the blue arrows indicate continuing significant differences between MAIT and non-MAIT cells at sequential time points.

Next, we examined cytokine expressing MAIT and CD8^+^ non-MAIT cells (CD8^+^CD4^−^MR1^−^) over the course of vaccination and infection following non-specific stimulation with PMA/Ionomycin. In blood, we observed an increase in cells expressing the antiviral cytokines, TNF-α (Fig. 2A) and IFN-γ (Fig. 2B), upon vaccination and infection compared to pre-levels, suggesting better functionality of both cell populations. In this and subsequent panels, cytokine expressing cells were normalized to matched cells obtained prior to immunization. Thus, the naïve level appears as a fold change of 1. Since in some cases pre-level cytokine expressing MAIT and CD8^+^ non-MAIT cells were different, use of fold change facilitated the comparison of the effect of vaccination and subsequent infection on both cell types. No difference between TNF-α- expressing MAIT and CD8^+^ non-MAIT cells was observed; however, more IFN-γ-expressing CD8^+^ non-MAIT cells compared to MAIT cells were seen over the course of the study. More MAIT cells expressing IL-17A (Fig. 2C) were also observed compared to CD8^+^ non-MAIT cells during the vaccination time period. Granzyme B expressing MAIT and non-MAIT cells fluctuated over the course of vaccination, however, after infection, more CD8^+^ non-MAIT cells expressed Granzyme B compared to MAIT cells (Fig. 2D). Both MAIT and non-MAIT cells expressing Ki-67, a proliferative marker, were elevated early in the vaccination regimen but overall comparable levels were observed in the two cell populations (Fig. 2E).

**Figure 3:**
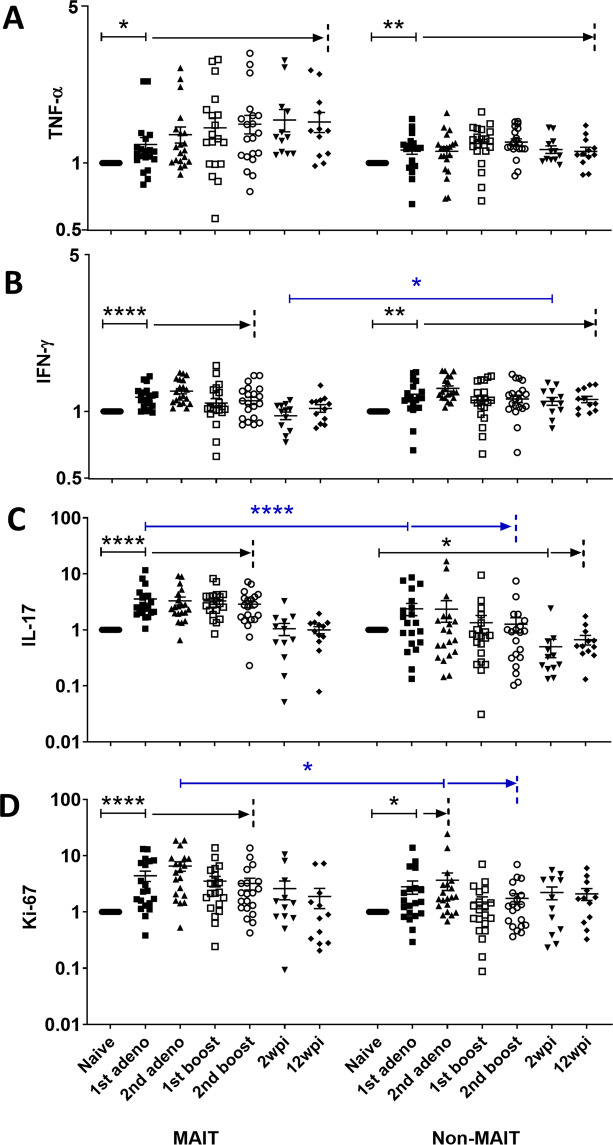
Comparison of fold change of cytokine expressing MAIT and CD8^+^ non-MAIT cell subsets in BAL. Fold change following PMA/Ionomycin stimulation of (**A**) TNF-α^+^, (**B**) IFN-γ^+^, (**C**) IL-17^+^ and (**D**) Ki-67-expressing MAIT and CD8^+^ non-MAIT cells compared to pre-samples (naïve) over the course of vaccination and infection. Data of (**A**–**D**) were analyzed by the Wilcoxon paired test. Horizontal and vertical bars denote mean and SEM. *p < 0.05, **p < 0.01, ****p < 0.0001. The black arrows indicate persistence of significant differences compared to the naïve sample up to the time point marked by the dashed line. For simplicity, p values for each comparison ranging from <0.05 to <0.0001 are not shown. Similarly, the blue arrows indicate continuing significant differences between MAIT and non-MAIT cells at sequential time points.

Similar to the pattern in blood, an increase in both MAIT and CD8^+^ non-MAIT cells in BAL expressing TNF-α was observed post-vaccination (Fig. 3A). An increase in IFN-γ-expressing MAIT and CD8^+^ non-MAIT cells was also observed upon vaccination, however, unlike the blood cells, IFN-γ-expressing MAIT cells decreased after infection (Fig. 3B). Both cell types exhibited comparable levels of TNF-α and IFN-γ expressing cells, except a higher level of IFN-γ expressing CD8^+^ non-MAIT cells was seen in the acute phase of infection. IL-17A expressing MAIT cells remained elevated over the course of vaccination along with a higher level compared to CD8^+^ non-MAIT cells (Fig. 3C). Again, unlike the pattern seen in blood cells, upon vaccination a higher level of Ki-67 expressing MAIT cells compared to CD8^+^ non-MAIT cells was observed (Fig. 3D). Granzyme B expressing cells were not assayed in BAL samples. Overall, the cytokine profile of MAIT cells in BAL differed from that of blood.

Taken together, the data suggest that vaccination increases cytokine expressing MAIT cells as well as their proliferation in blood and lung. Unlike blood, IFN-γ-expressing MAIT cells in BAL samples decreased after SIV infection, suggesting that these cells are more affected in the tissue compared to circulation. However, TNF-α-expressing cells were maintained in both blood and BAL after infection, suggesting that MAIT cells retain some functionality even after SIV infection.

### MAIT cells express cytokines and chemokines which possess the capacity to attract and help B cells

**Figure 4:**
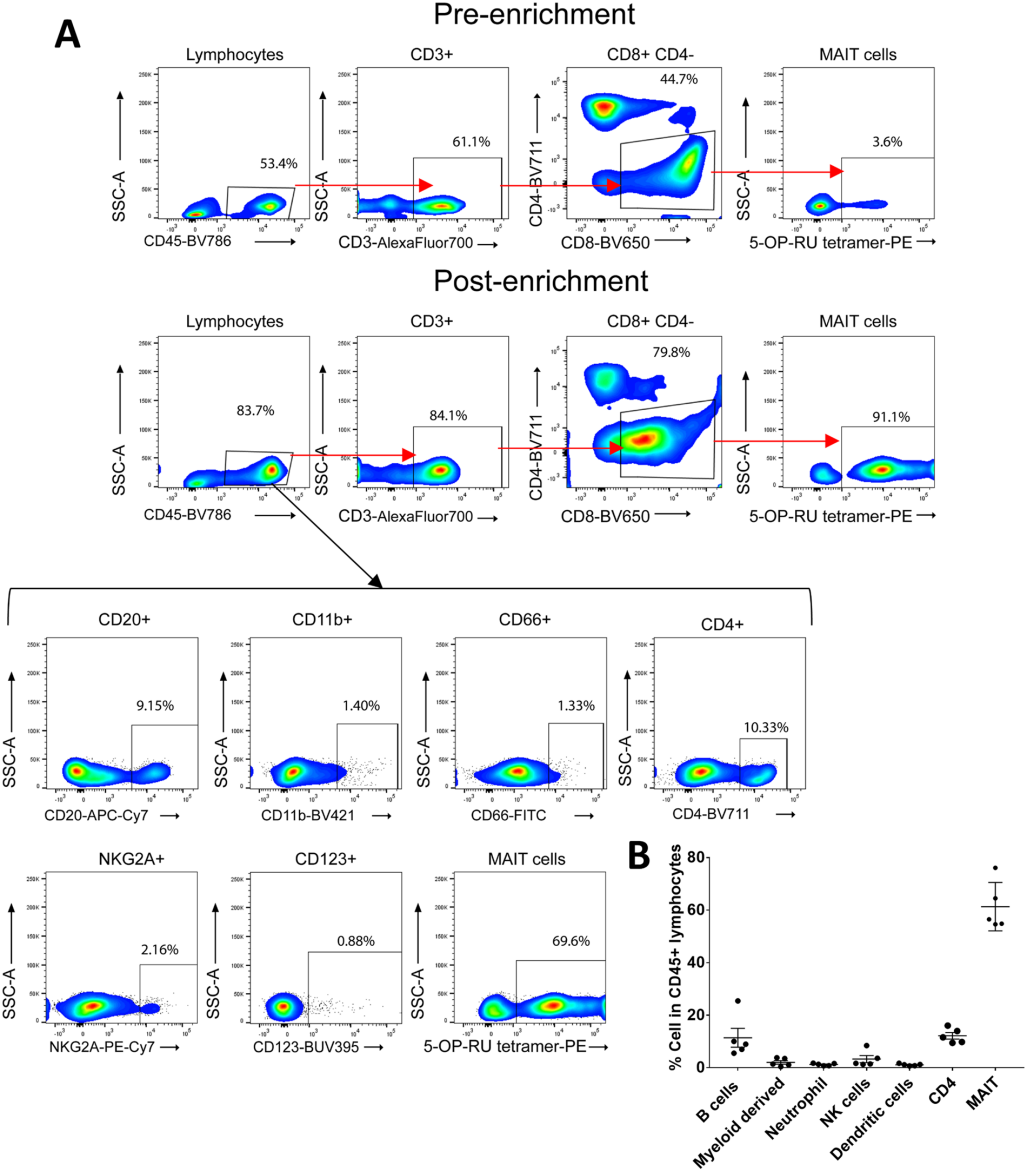
Determination of the purity of enriched MAIT cells. (**A**) Gating strategy to determine cell population subsets in the magnetically enriched MAIT cells. (**B**) Frequency of cell types in CD45^+^ lymphocytes.

**Figure 5:**
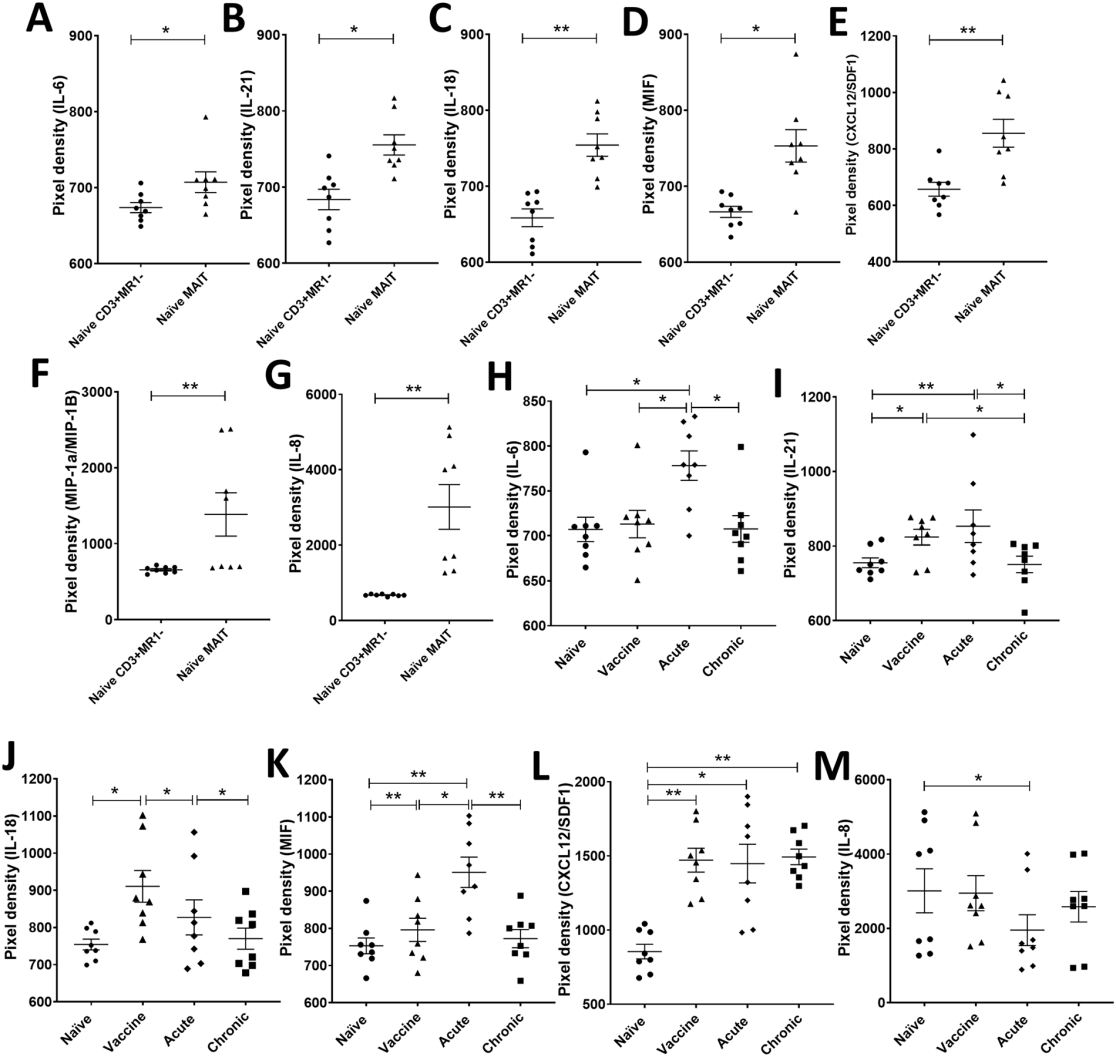
Evaluation of cytokines/chemokines in culture supernatants of enriched MAIT cells and CD3^+^MR1^−^ cells. (**A**–**G**) Comparison of IL-6, IL-21, IL-18, MIF, CXCL12/SDF1, MIP1α/1β and IL-8 expression in the α-CD3/α-CD28 stimulated 24-hour culture supernatants of MAIT cells and CD3^+^MR1^−^ cells from naïve macaques. (**H**–**M**) Cytokines/chemokines in the culture supernatant of enriched MAIT cells from naïve, vaccinated, acute and chronically SIV-infected animals. Data of (**A**–**M**) were analyzed by Wilcoxon paired test. Horizontal and vertical bars denote mean and SEM. *p < 0.05, **p < 0.01.

We next turned to the question of whether MAIT cells can provide help to B cells in rhesus macaques and whether vaccination and SIV infection affects this capability. We enriched naïve MAIT and CD3^+^MR1^−^ cells from PBMC as described in Materials and Methods. BAL cells were limited and not available in sufficient numbers for this experiment. The majority of the purified CD45^+^ lymphocytes were 5-OP-RU tetramer positive (Fig. 4A). In addition, we observed 5–25% CD20^+^ B cells, <4% CD11b^+^ myeloid derived cells, <2% CD66^+^ neutrophils, 1%–8% NKG2A^+^ NK cells, <2% CD123^+^ dendritic cells, and 9%-16% CD4^+^ T cells present in the magnetically separated CD45^+^ lymphocyte population (Fig. 4A,B). We assessed the cytokine secretion of the magnetically enriched naïve MAIT and CD3^+^MR1^−^ cells via cytokine array following T cell-specific stimulation for 24 hours with α-CD3 and α-CD28 to avoid stimulation of the small populations of non-T-cells in the enriched samples. Out of 36 cytokines/chemokines assayed, 7 were detected in MAIT cell culture supernatants, including IL-6, IL-21, IL-18, macrophage migration inhibitory factor (MIF), CXC chemokine ligand 12/stromal cell-derived factor (CXCL12/SDF-1), macrophage inflammatory protein-1α/β (MIP-1α/β) and IL-8 (Fig. 5A–G). These cytokines/chemokines potentially contribute to B cell activation, proliferation, differentiation and migration as addressed further in the Discussion section. The autologous stimulated CD3^+^MR1^−^ cells secreted lower amounts of the same cytokines/chemokines compared to MAIT cells (Fig. 5A–G). Taken together, the data suggested that MAIT cells have a greater potential to influence B cells compared to CD3^+^MR1^−^ cells.

We next analyzed supernatants from magnetically enriched α-CD3/α-CD28-stimulated MAIT cells obtained from naïve, vaccinated (post-2^nd^ Ad5hr-recombinant immunization), acute (2 wpi) and chronically (12 wpi) SIV infected animals in order to examine the effect of Ad-based vaccination and SIV infection on the ability of MAIT cells to modulate B cells. Secretion of IL-21 (Fig. 5I), MIF (Fig. 5K), CXCL12/SDF-1 (Fig. 5L) and IL-18 (Fig. 5J) increased upon vaccination; the first three increased further compared to naïve levels after acute infection. They all returned to naive levels after chronic infection with the exception of CXCL12/SDF-1, which remained elevated. IL-6 increased (Fig. 5H) while IL-8 decreased (Fig. 5M) after acute infection although both returned to the pre-level after chronic infection. MIP-1α/β secretion remained unchanged over the course of vaccination and infection (data not shown). Thus, vaccination as well as acute infection differentially effected cytokine/chemokine secretion of MAIT cells, resulting in differing potentials for attracting and helping B cells.

### Influence of MAIT cells on antibody production

**Figure 6:**
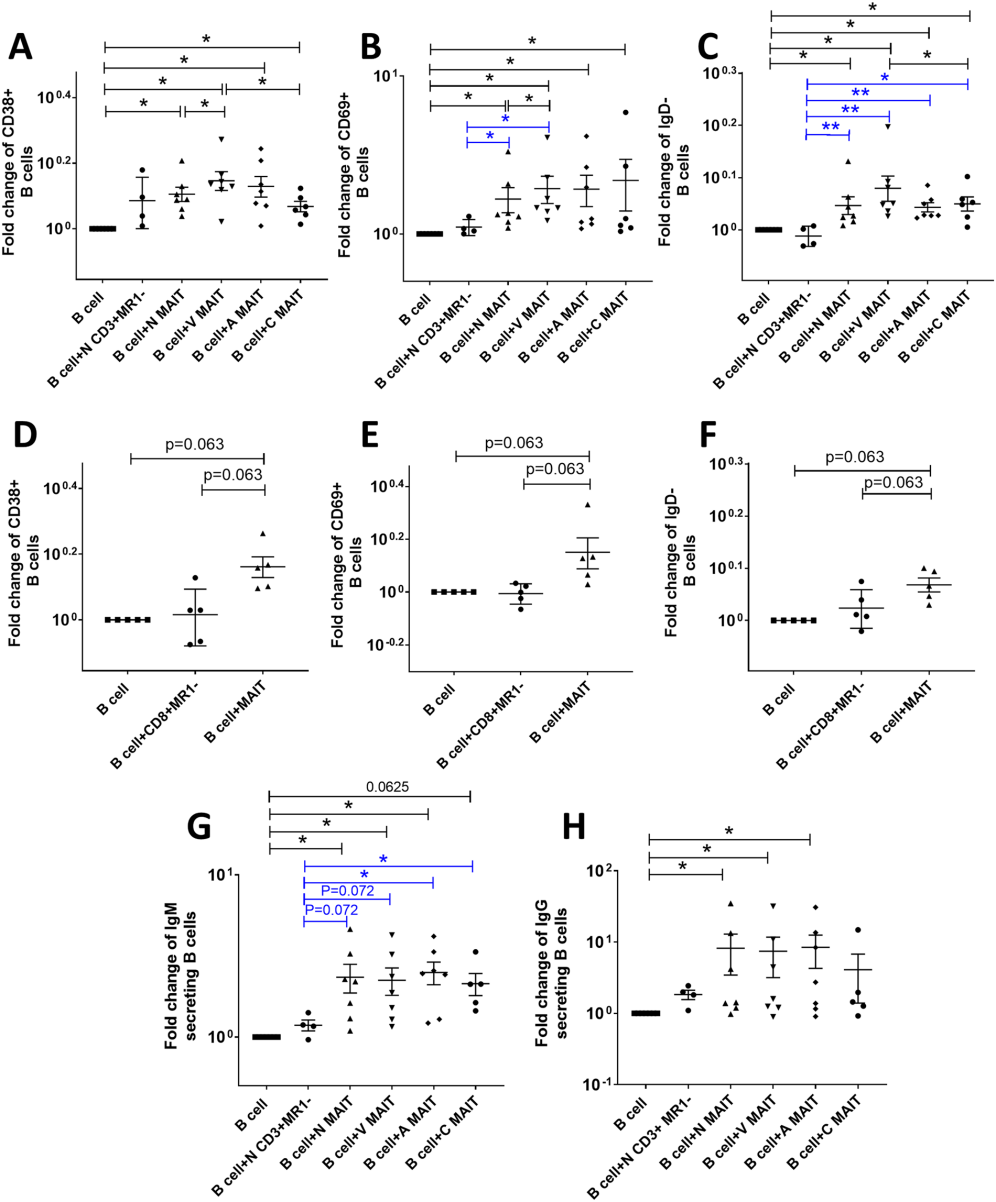
Influence of MAIT cells on B cell activation and antibody secretion. Following stimulation with enriched (**A–C**) or sorted (**D**–**F**) MAIT cell supernatants, naïve B cells exhibit changes in expression of (**A,D**) CD38, (**B,E**) CD69 and (**C,F**) IgD. B cells stimulated with enriched MAIT cell supernatants from naïve, vaccinated, acute and chronically infected macaques were compared with B cells cultured alone or with B cells cultured with supernatant from naïve enriched CD3^+^ MR1^-^ cells (**A**–**C**). B cells cultured with sorted MAIT cell supernatants were compared with B cells cultured alone or with B cells cultured with sorted CD8^+^MR1^−^ cells (**D**–**F**). Enriched MAIT cell supernatant-stimulated B cells secrete (**G**) IgM and (**H**) IgG. Data are expressed as fold change calculated based on respective B cell alone data. Data of (**A**–**H**) were analyzed by the Wilcoxon paired test or Mann–Whitney U test. Horizontal and vertical bars denote mean and SEM. *p < 0.05.

To further investigate the potential influence of MAIT cells on B cells, the α-CD3/α-CD28-stimulated enriched MAIT cell supernatants were collected and cultured with autologous naïve B cells. Similarly, stimulated CD3^+^MR1^−^ cell supernatants were used as controls. The changes in B cells upon stimulation with naïve, vaccinated, acute and chronically infected macaque MAIT cell supernatants were compared with B cells cultured alone or with B cells cultured in the presence of CD3^+^MR1^−^ culture supernatant by flow cytometry. The gating strategy is shown in Fig. S5. After 7 days of culture, B cells expressing the activation and plasma cell differentiation marker, CD38, (Fig. 6A) and the activation marker, CD69, (Fig. 6B) together with the loss of IgD expression, an indication of class switching (Fig. 6C), were elevated compared to B cells cultured alone. No difference in levels of B cells expressing CD38 was observed when stimulated with MAIT or CD3^+^MR1^−^ cell supernatant (Fig. 6A), suggesting both cell types have the ability to influence B cells. However, B cells expressing CD69 (Fig. 6B) and loss of IgD (Fig. 6C) were elevated upon stimulation with MAIT supernatant but not CD3^+^MR1^−^ supernatant, suggesting MAIT cells have greater capacity for activating B cells and promoting class switching. Culture of vaccinated MAIT cell supernatant with B cells resulted in higher levels of CD38 and CD69 expressing cells compared to culture with naïve MAIT cell supernatant, suggesting a greater potential to activate B cells to secrete antibody. Following infection, MAIT cell supernatants exhibited similar effects as MAIT cells from vaccinated macaques, although levels of CD38 expressing cells declined somewhat (Fig. 6A) and loss of IgD expression was not as great (Fig. 6C). However, post-infection MAIT cells appeared to retain some capacity to modulate B cells.

To further confirm the data, MAIT cells and CD8^+^ non-MAIT (CD3^+^CD4^−^CD8^+^MR1^−^) cells from PBMCs of 5 post-2^nd^ boost animals were sorted to 95% purity by flowcytometry and stimulated with α-CD3/α-CD28. The supernatants were collected, and autologous B cells were cultured in the presence of the supernatants. The B cells showed a trend of higher levels of CD38 (Fig. 6D), CD69 (Fig. 6E) and loss of IgD expressing cells (Fig. 6F) when cultured in the presence of sorted MAIT cell supernatant compared to that when cultured alone or in the presence of sorted CD8^+^ non-MAIT cell supernatant. The same result was seen when cell frequencies were plotted instead of fold change values (Fig. S6).

Next, we evaluated antibody secretion by B cells in the presence or absence of various enriched MAIT and CD3^+^MR1^−^ cell supernatants. MAIT cell supernatant induced naïve B cells to secrete significantly higher levels of IgM (Fig. 6G) and IgG (Fig. 6H) compared to B cells cultured alone. A trend of higher IgM secretion was observed by MAIT cell supernatant compared to CD3^+^MR1^−^ cell supernatant (Fig. 6G). No such difference was observed for IgG secretion, however (Fig. 6H). Due to limitation of samples we were not able to measure IgA levels in the culture supernatants. Although the surface marker analysis suggested a potential for the supernatant of vaccinated MAIT cells to induce higher antibody levels, no difference was observed compared to naïve MAIT cell supernatant for either IgM or IgG production. Note, however, that naïve B cells were used in this experiment, so SIV-specific antibody would not be induced. Taken together, the data suggested that MAIT cells have the potential to enhance B cell activation and secretion of antibody.

### MAIT cells modulate generation of memory B cells

**Figure 7:**
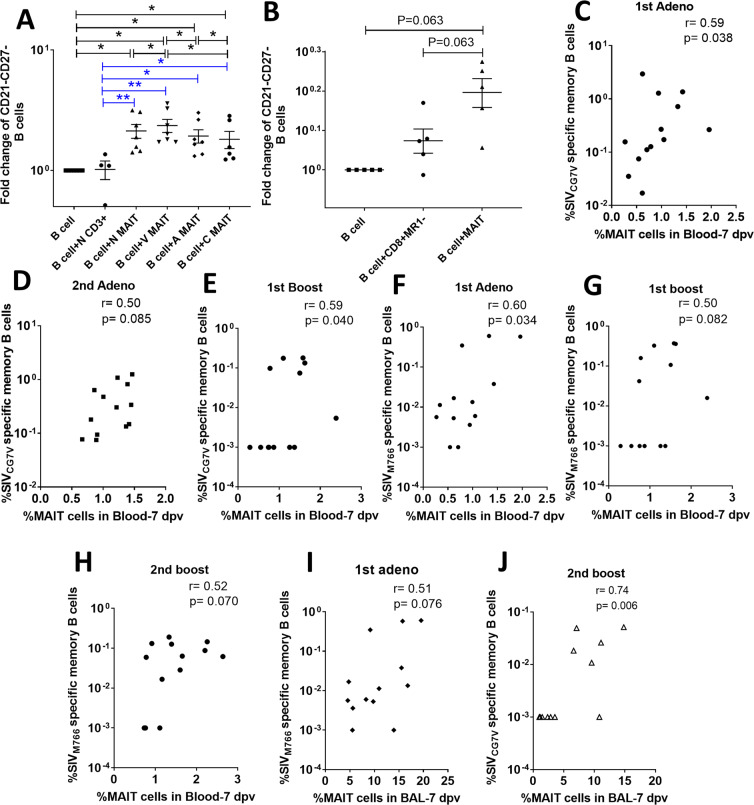
MAIT cells drive B cells toward tissue resident memory B cell formation. (**A,B**) The fold change (calculated based on respective B cell alone data) of CD21^−^CD27^−^ TLM B cells of naïve B cells stimulated with enriched (**A**) or sorted (**B**) MAIT cell supernatants *in vitro*. (**C**–**J**) Association or trend of association of the frequency of MAIT cells in blood or BAL 7 dpv with SIV Env-specific memory B cells in rectal mucosa 21 dpv. Data of (**A,B**) were analyzed by the Wilcoxon paired test. Horizontal and vertical bars denote mean and SEM. *p < 0.05. Data of (**C**–**J**) were analyzed by the Spearman correlation test.

To pursue the MAIT cell-B cell interaction, we examined memory B cell populations following stimulation of naïve B cells with enriched MAIT cell supernatants. Based on CD21 and CD27 expression, 4 populations have been previously described in macaque peripheral blood: naïve (CD21^+^CD27^−^), resting memory (CD21^+^CD27^+^), activated memory (CD21^−^CD27^+^), and tissue-like memory (TLM) (CD21^−^CD27^−^)^32^. Here, a downregulation of CD21 and CD27 expression was observed on B cells cultured with MAIT cell supernatant leading to an increase in TLM cells compared to B cells cultured alone or with CD3^+^MR1^−^ cell supernatant (Fig. 7A). Vaccinated MAIT cell supernatant showed greater potential to generate TLM B cells compared to stimulation with other supernatants. We repeated the experiment using the flowcytometry-sorted cells and again observed that B cells showed a higher frequency of the CD21^−^CD27^−^ phenotype in the presence of MAIT cell supernatant compared to the CD8^+^MR1^−^ supernatant (Fig. 7B). TLM B cells have been shown to be the majority population in mucosal tissues of rhesus macaques^32^. Although in humans, PB and plasma cells (PC) are characterized by expression of CD27, macaque TLM B cells have been shown to secrete antibody, clearly indicating they are PB/PC^33^. In fact, macaque mucosal memory B cells lack CD27 expression with only naïve and TLM B cell populations observed^34^.

To understand these *in vitro* data, we correlated the frequencies of MAIT cells with rectal Env-specific memory B cells. Positive correlations or trends of positive correlations were observed between the frequency of blood MAIT cells 7 days post vaccination (dpv) with rectal SIV_CG7V_ Env-specific (Fig. 7C–E) and SIV_M766_ Env-specific (Fig. 7F–H) memory B cells 21 dpv over the course of vaccination; however, such correlations were not observed for the SIV_CG7V_ Env-specific response after the 2^nd^ boost and the SIV_M766_ Env-specific response after the 2^nd^ adeno. After the 1^st^ adeno a trend of positive association was observed between BAL MAIT cells and rectal SIV_M766_ Env-specific memory B cell responses (Fig. 7I). Furthermore, after the 2^nd^ boost a strong positive correlation was observed between BAL MAIT cells and rectal SIV_CG7V_ Env-specific memory B cell responses (Fig. 7J). Together these data suggested that MAIT cells have the potential to generate tissue resident antigen specific memory B cells.

**Figure 8:**
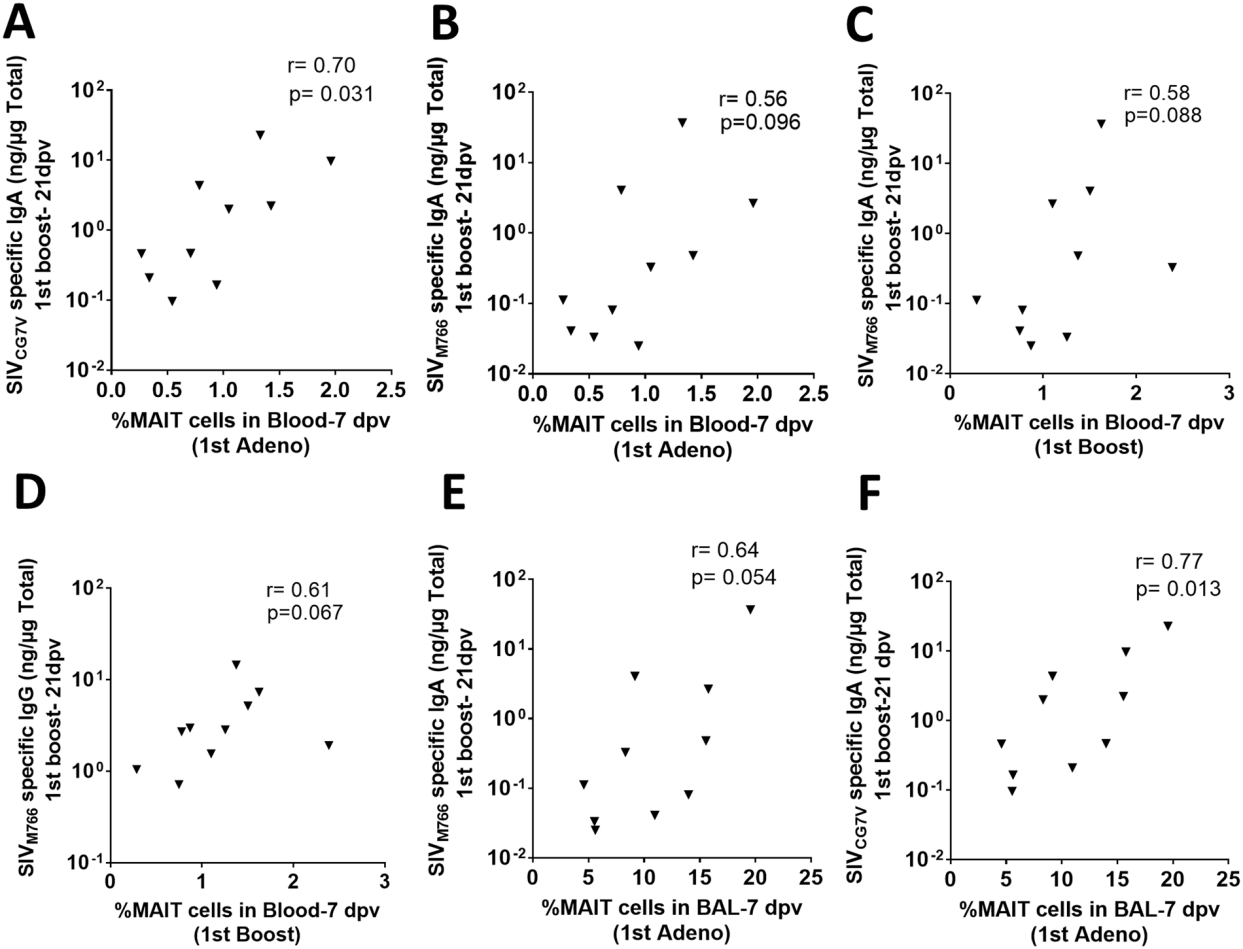
MAIT cell frequency in blood or BAL influences antigen specific antibody secretion in rectal mucosa. (**A**–**F**) Association or trend of association of the frequency of MAIT cells in blood or BAL 7 dpv with antigen specific antibody secretion in the rectal mucosa. Data of (**A**–**F**) were analyzed by the Spearman correlation test.

To further confirm the data, we determined the correlation between the frequency of MAIT cells and SIV Env-specific antibody levels in rectal secretions. The blood MAIT cell frequency 7dpv with the 1^st^ adeno, positively correlated with rectal SIV_CG7V_ Env-specific IgA 21 days after the 1^st^ boost (Fig. 8A) and tended to correlate with SIV_M766_ Env-specific IgA at the same time point (Fig. 8B). MAIT cells 7 days after the 1^st^ boost also tended to correlate with rectal SIV_M766_ Env-specific IgA and IgG antibody levels 21 days after the 1^st^ boost (Fig. 8C,D). The BAL MAIT cell frequency 7 days post-1^st^ adeno showed a trend of positive correlation with SIV_M766_ Env-specific IgA (Fig. 8E) and a positive correlation with SIV_CG7V_ Env-specific IgA 21 days post-1^st^ boost (Fig. 8F). Taken together, MAIT cells *in vitro* showed the potential to generate TLM B cells irrespective of the naïve, vaccination or infection status of the animals. However, correlation analyses of *ex vivo* MAIT cells and SIV-specific rectal antibodies suggested that following vaccination MAIT cells have a greater potential to influence the generation of TLM B cells and antibody-secreting B cells compared to naïve MAIT cells.

## Discussion

As a component of the innate immune system, MAIT cells respond quickly to infectious agents but have also exhibited activation and increased frequencies in response to vaccination in humans^16,18^, mice^15^ and macaques^19^. The effect of vaccination on MAIT cells in the SIV rhesus macaque model has not yet been described. In the current longitudinal study, we evaluated effects of our vaccine regimen composed of mucosal priming with replicating Ad5hr-SIV recombinants and boosting with SIV Env protein in alum adjuvant. We observed that the vaccine regimen increased MAIT cell frequency in blood over the entire course of immunization (Fig. 1) and boosted cytokine-expressing MAIT cells in both blood and BAL (Figs. 2–3). However, no difference in these parameters was observed between the vaccine and control group, which received empty Ad5hr vector and alum only, suggesting that the observed MAIT cell responses were due to the vaccine vector and adjuvant rather than the SIV antigens. We have previously observed a lack of difference in responses of other innate immune cells including neutrophils^35^, dendritic cells^36^, and NK/innate lymphoid cells^37^ between vaccine and control groups when both have received the Ad vaccine vector and adjuvant.

Compared to naïve subjects a lower frequency of MAIT cells has been observed in HIV infection^8–11^ as well as in chronic SIV infection of rhesus macaques^12^; however, direct pre- to post- HIV/SIV infection comparisons were not reported. In a longitudinal SIV/SHIV pigtail monkey model no evidence of MAIT cell depletion in early infection was observed nor were MAIT cells reduced in the periphery in long-term chronic infection^13^. In the current longitudinal study in rhesus macaques neither MAIT cells in blood or the lung exhibited decreased frequencies compared to pre-immunization levels in the acute or early chronic phase of SIV infection. This difference from the cross-sectional study of Vinton *et al*.^12^ may reflect differences in the macaques studied. In Vinton *et al*. 7 out of 29 macaques showed AIDS like symptoms, whereas in our study none of the macaques were symptomatic and except for 2 macaques where CD4 counts were not available, all possessed greater than 10% absolute CD4^+^ T cells 14 to 19 wpi. Whether the preservation of MAIT cells observed here is a residual effect of vaccination will require further study.

Different subsets of T cells^20–23^ have been shown to provide help to B cells. *Ex vivo* analysis of human MAIT cells has shown that they can secrete cytokines associated with B cell help, including IL6, IL-10 and IL-21, and are associated with increased PB frequencies and antibody production^23^. Here we report that T cell-specific-stimulated rhesus macaque MAIT cells also secrete several cytokines/chemokines (IL-21, IL-6, CXCL12/SDF1, MIP-1α/MIP-1β, MIF, IL-18, and IL-8) known to modulate B cells. Studies in mice and humans have shown IL-21 is crucial for generation of T_FH_ cells and formation of germinal centers, thus contributing to B cell growth, survival, and differentiation of memory B cells into PC, and therefore antibody production^38^. IL-6, first shown to enhance antibody production^39^, is also important for differentiation of B cells into PC^40^. This proinflammatory cytokine acts indirectly by induction of IL-21 production by CD4^+^ T cells^41^. CXCL12/ SDF-1 is necessary for B cell development and colonization of the bone marrow and helps maintain early B cell precursors in cellular niches required for further development^42^. The chemokines MIP-1α/MIP-1β are associated with B cell proliferation^43^ and also stimulate mucosal and systemic antigen–specific antibody responses as well as cellular responses^44^. MIF maintains the mature B cell population by initiating a survival pathway for rescue of mature B cells from death^45^. IL-18 has been shown to increase production of Th2 cytokines^46,47^. IL-8 is an important inflammatory mediator that induces directional migration and recruitment of B lymphocytes^48^. Thus, the cytokines/chemokines secreted by MAIT cells in the current study show an overall B cell help potential including activation, proliferation, differentiation, and regulation. Of note, secretion of IL-21, MIF, IL-18 and CXCL12/SDF1 by MAIT cells following vaccination was elevated compared to pre-immunization MAIT cells, suggesting a potential benefit of vaccination on this cell population.

The magnetically enriched MAIT cells contained small populations of other cells including NK cells, dendritic cells, myeloid derived cells, neutrophils, B cells, and CD4^+^ T cells (Fig. 4), although MAIT cells comprised about 61% of the cells. These minor cell populations can express some of the relevant cytokines/chemokines involved in B cell help. For example, IL-18 has been shown to be expressed by macrophages and dendritic cells^49^, IL-6 by T cells, B cells and macrophages^50^, IL-21 by T cells and NKT cells^51^, MIF by cells of the monocyte/macrophage lineage^52^, MIP-1α/1β by macrophages, NK cells, and T cells^53^, IL-8 by monocytes/macrophages and neutrophils^54^, and CXCL12/SDF-1 by monocytes^55^. CD4^+^ T cells can certainly provide B cell help, including a small population of CXCR5^+^CD4^+^ circulating T_FH_ cells present in blood^56^. Thus, we cannot exclude the possibility that the small populations of other cell types contributed to the secretion of these cytokines/chemokines. However, the enriched cells were stimulated with anti-CD3 and anti-CD28 antibodies, providing T cell-specific stimulation, unlikely to stimulate non-T cell populations. Since our goal was to determine specific cytokine release by MAIT and non-MAIT cells upon stimulation, we avoided using cytokine-mediated stimulation, such as IL-12/18, of these cells. Moreover, the control CD3^+^MR1^−^ cell cultures exhibited lower levels of the cytokines/chemokines associated with B cell help compared to the MAIT cell cultures, minimizing the effect of the CD4^+^ T cells as well as the other lower frequency cell populations. Further, when B cells were cultured in the presence of supernatants of MAIT cells sorted to 95% purity, results similar to those of the enriched MAIT cells were observed, further supporting the role of MAIT cells in provision of B cell help.

The potential B cell help provided by the cytokines/chemokines in the MAIT cell supernatants was pursued by analysis of the B cells co-cultured with the supernatants. We observed higher levels of B cells expressing CD69 and CD38, suggesting activation, and increased IgD^−^ B cells suggesting class switching. The supernatants of B cell cultures also showed secretion of IgG and IgM. Furthermore, the MAIT cell frequency post-1^st^ adeno in both blood and BAL correlated or showed a trend of correlation with SIV-specific antibody levels in the rectal secretions. Correlative trends were also observed between MAIT cell frequencies post-1^st^ boost in blood and post-1^st^ boost antibody levels in rectal secretions, suggesting that MAIT cells might indeed influence antibody secretion *in vivo*. Although surface marker analysis of B cells suggested a more activated B cell population for vaccinated animals, no difference in antibody production was observed upon stimulation of MAIT cells from naïve, vaccinated, acute and chronically SIV infected animals (Fig. 6G,H). Since naïve B cells were used in this *in vitro* experiment, antigen-specific antibody was not assayed. Vaccinated MAIT cells might influence a robust antigen-specific antibody response, which should be addressed in future studies.

Coculture of B cells with MAIT cell supernatants led to increased TLM B cells (Fig. 7A,B). These cells have been described as an exhausted dysfunctional memory cell subset that expresses inhibitory receptors in HIV viremic individuals^57^. However, as mentioned above, TLM cells in rhesus macaques secrete antibody despite the absence of CD27^33^. Whether the CD21^−^CD27^−^ TLM population arises in part from CD27 shedding is not known. However, macaque mucosal memory B cells lack CD27 expression, while the mucosal TLM subset has been shown to secrete IgA and express the J chain^34^. Thus, the cytokines/chemokines present in MAIT cell supernatants effectively enhanced the ability of macaque B cells to develop into PC. Furthermore, the frequency of MAIT cells 7 days post 1^st^ adeno vaccination both in blood (Fig. 8A,B) and BAL (Fig. 8E,F) correlated or tended to correlate with SIV Env-specific IgA responses in rectal secretions 21days post 1^st^ boost. Similar trends of correlation were also observed between MAIT cell frequency in blood and SIV Env-specific antibody responses in rectal secretions after the 1^st^ boost vaccination (Fig. 8C,D), suggesting that MAIT cells influenced the development of antigen-specific antibody responses. Notably, statistically significant correlations were observed between blood and BAL MAIT cells and SIV_CG7V_ Env-specific rectal IgA antibodies (Fig. 8A,F) while only correlative trends were seen with SIV_M766_ Env-specific antibodies (Fig. 8B–E). The SIV_CG7V_ is more closely related to SIV_smH4_, the SIV strain used in the Ad5hr-SIV*env* recombinant priming immunogen, than SIV_M766_, providing further suggestive evidence of a vaccine effect. We did not assay adenovirus-specific antibody, although Ad-specific antibodies have been observed upon vaccination^58,59^. Here our interest was in development of SIV-specific, not vector-specific antibody by vaccination. However, as our data suggest MAIT cells provide help in generating SIV-specific humoral responses, it is likely that they also play a role in Ad-specific antibody induction.

We observed that over the course of immunization the frequency of MAIT cells in blood and BAL correlated with SIV-specific memory B cell responses (Fig. 7C–J). We did not evaluate migration markers on MAIT cells which might have indicated preferences for movement of these cells to specific tissues. Since all the experiments were done *in vitro*, it is difficult to identify sites where B cell-MAIT cell interactions might have led to provision of B cell help. Whether MAIT cells provide help to B cells in B cell follicles remains unexplored. MAIT cells are found most abundantly in the liver (up to 40% of resident T cells) and also in mucosal tissues, such as the lung and gut^60^. Since we observed positive correlations between memory B cells in rectal tissue and MAIT cell frequencies in blood and BAL (Fig. 7C–J), it is possible that interactions between MAIT cells and B cells take place in the gut. As liver has been identified as a site of IgA production by B cells^61^ and it receives the majority of its blood supply from the gut, the liver might be another site of B cell-MAIT cell interaction. Further studies are needed to answer this question.

Overall, this longitudinal study has shown that a SIV vaccine regimen increased MAIT cell frequency and cytokine/chemokine expression. SIV infection affected the tissue resident MAIT cell population more potently compared to circulating MAIT cells. Further, our results indicated that MAIT cells have the potential to help B cells generate TLM cells and to activate B cells to secrete antibodies. Our results suggest that vaccines that stimulate MAIT cells, which subsequently enhance B cell responses, are desirable.

## Materials and Methods

### Study animals and immunization

Female rhesus macaques were maintained at the National Cancer Institute Animal Facility under the guidelines of the Association for the Assessment and Accreditation of Laboratory Animal Care and according to the recommendations of the *Guide for the Care and Use of Laboratory Animals*. The protocol (VB027) and procedures were approved by the NCI Animal Care and Use Committee prior to study initiation. Briefly, macaques (n = 13) were immunized at week 0 (intranasally and orally) and week 13 (intratracheally) with replicating Adenovirus type 5 host range mutant (Ad5hr) recombinants separately expressing SIV_smH4_ Env, SIV_239_ Gag, and SIV_239_ Nef followed by boosting with SIV_M766_ and SIV_CG7V_ gp120 in alum hydroxide adjuvant intramuscularly at weeks 26 and 38^62^. The control group (n = 7) received Ad5hr empty vector at a dose equivalent to the Ad-SIV recombinants and alum only. Up to 15 low-dose SIV intravaginal challenges were given weekly until infection occurred as determined by viral loads of ≥50 SIV RNA copies/ml plasma, determined by droplet digital PCR (Chung *et al*., in preparation).

### Sample collection

Blood and BAL samples were obtained from macaques before, at day 7 after each immunization and at 2 and 12 wpi. PBMCs were processed by density gradient centrifugation over Ficoll as described^63^. BAL fluids were centrifuged and removed. PBMCs and cells from BAL samples were frozen in FBS containing 10% DMSO and stored in liquid nitrogen until use.

### Flow cytometric detection of circulatory and mucosal MAIT cells

PBMC and BAL cells were stained with 5-OP-RU tetramer or 6-FP control tetramer conjugated with PE (NIH Tetramer Core Facility, Emory University, Atlanta, GA) for 30 minutes at room temperature (RT) at a 1:100 dilution (v/v). Cells were washed and stained with aqua Live/Dead viability dye (Invitrogen, Carlsbad, CA) at RT for 15 min in PBS, washed with FACS wash (PBS + 2% FBS), and surface stained with the following anti-human fluorochrome-conjugated mAbs known to cross-react with rhesus macaque antigens: PE CF594 anti-CD3 (SP34-2), BV711 anti-CD4 (L200), BV650 anti-CD8 (RPA-T8) all from BD Biosciences, San Jose, CA. At least 500,000 singlet events were acquired on a SORP LSR II (BD Biosciences) and analyzed using FlowJo Software (FlowJo, Ashland, OR). For all samples, gating was established using a combination of isotype and fluorescence-minus-one (FMO) controls.

### Cytokine staining of stimulated MAIT cells

To measure cytokine production of MAIT cells upon nonspecific stimulation, cells were stimulated with PMA/Ionomycin in the presence of BD GolgiPlug and BD GolgiStop. Following incubation for 12–14 hours at 37 °C in the presence of 5% CO_2_ the cells were stained with Live/Dead aqua dye followed by surface staining and permeabilization with Foxp3/Transcription Factor Staining buffer (Invitrogen Life Technologies, Carlsbad, CA). Intracellular staining was performed with BV421 anti–IFN-γ (B27), Alexa fluor 700 anti–Ki67 (B56), FITC anti–GranB (GB11) from BD Biosciences, San Jose, CA; BV605 anti–TNF-α (MAb11) from BioLegend, San Diego, CA; and PerCP-Cy5.5 anti–IL-17A (eBio64DEC17) from Invitrogen Life Technologies, Carlsbad, CA. Acquisition and analysis followed the same protocol as for MAIT cell detection. Stimulation-specific cytokine responses were calculated by subtracting the % unstimulated response from % stimulated response. Fold change of cytokine expression was determined based on respective expression of the pre-samples.

### Isolation of PBMC cell subsets

Viably frozen PBMCs obtained from 8 macaques prior to immunization, post-2^nd^ Ad5hr-recombinant immunization, and 2 wpi and 12 wpi were thawed and stained (1 ul 5-OP-RU tetramer-PE/1 million cells) for 30 minutes at RT, magnetically labeled with anti-PE magnetic beads (Miltenyi Biotec), and positively isolated using a magnetic column. This subset was considered enriched MAIT cells (Fig. 4). MR1^−^ cells were labeled with anti-CD3 magnetic beads (Miltenyi Biotec), isolated using a magnetic column and considered CD3^+^MR1^−^ T cells. MR1^−^CD3^−^ cells were labeled with anti-CD19 magnetic beads (Miltenyi Biotec), isolated using a magnetic column and considered B cells. To check the purity of the enriched MAIT cells, PBMC obtained prior to immunization from 5 additional macaques were stained with 5-OP-RU tetramer conjugated with PE (NIH Tetramer Core Facility) for 30 minutes; followed by Alexa700 anti-CD3 (SP34-2), BV711 anti-CD4 (L200), BV650 anti-CD8 (RPA-T8), APC-Cy7 anti-CD20 (2H7), BUV395 anti-CD123 (7G3), BV421 anti-CD11b (ICRF44) BV786 anti-CD45 (D058-1283) all from BD Biosciences, FITC anti-CD66_abce_ (TET2) from Miltenyi Biotec, PE-Cy7 anti-NKG2A (Z199) from Beckman Coulter. The cells were acquired on a Symphony (BD Biosciences) and analyzed as described above.

### Cell sorting

PBMCs obtained following the 2^nd^ boost immunization were stained with 5-OP-RU tetramer conjugated with PE (NIH Tetramer Core Facility) for 30 minutes, followed by Alexa700 anti-CD3 (SP34-2), APC anti-CD4 (L200), FITC anti-CD8 (RPA-T8), and PE-Cy7 anti-CD20 (2H7) (BD Biosciences). Aqua Live/Dead viability dye was used to exclude dead cells. After staining, cells were washed, passed through a 40-mm cell strainer, and sorted on an Astrios EQ flow cytometer. Two groups of live cells were sorted (CD3^+^CD4^−^CD8^+^MR1^+^, and CD3^+^CD4^−^CD8^+^MR1^−^) to a purity of 95%. Live B cells were sorted using PE-Cy7 anti-CD20 (2H7) (BD Biosciences) from pre-vaccinated animals to a purity of 95%.

### Influence of MAIT cells on B cell function

MAIT or CD3^+^MR1^−^ cells (25000) were stimulated with anti-CD3 (5 µg/ml) and anti-CD28 (10 µg/ml) antibodies for 24 hours. Supernatants were collected and used in B cell stimulation assays or for determination of cytokine profiles. B cells from naïve animals were isolated using the above protocol and placed in a 96 well U-bottom plate (250,000 cells/well) in 100 µl of R10. Additional autologous cell supernatants (100 μl) were added to each well from the following subsets: naive CD3^+^MR1^−^ cells, naive MAIT cells, vaccinated (post-2^nd^ Ad5hr-recombinant immunization) MAIT cells, acutely infected (2wpi) MAIT cells, and chronically infected (12wpi) MAIT cells. As a control, B cells alone were cultured in the presence of R10. The cultures were maintained for 7 days. Supernatants were collected and stored at −80 °C for later determination of antibody levels. The cells were analyzed by flowcytometry following staining with Live/Dead aqua dye, Alexa flour 700 anti-CD3 (SP34-2), BV711 anti-CD69 (FN50), BV605 anti-CD21 (B-ly4) from BD Biosciences, San Jose, CA; BV650 anti-CD20 (2H7), APC anti-CD27 (O323) from Invitrogen Life Technologies, Carlsbad, CA; FITC anti-CD38 (AT-1) from Stemcell Technologies, Vancouver, Canada; and Goat Anti-Human IgD-TXRD from Southernbiotech, Birmingham, AL. Acquisition and analysis followed the same protocol as for MAIT cells. The fold change of the different cellular markers was calculated based on marker expression by B cells cultured alone.

### Determination of cytokine/chemokine levels

The relative levels of 36 cytokines and chemokines in the supernatants of B cell cultures were analyzed using a human cytokine array (Catalog # ARY005, R&D System, Minneapolis, MN, USA), according to the manufacturer’s instructions^64^. Briefly, culture supernatants (100 μl) collected after centrifugation were added to dot blots onto which capture Abs had been spotted in duplicate. After incubation with the secondary Ab mixture, the resultant signals were detected using a Bio-Rad image analyzer. The intensity of the spots was quantified using ImageJ software.

### Quantification of IgG and IgM level in culture supernatant

Frozen B cell culture supernatants were thawed and passed through 5 µm polyvinylidene fluoride (PVDF) centrifugal filters (Merck Millipore, IRL) to remove any non-soluble particles. 96-well ELISA plates were coated overnight at 4 °C with 5 ug/well of goat anti-monkey IgG (Alpha Diagnostic) or mouse monoclonal anti-monkey IgM. Plates were blocked using 1% BSA solution (SeraCare). Supernatants diluted 5-fold with PBS were added in duplicate and incubated for 1 h at 37 ^o^C. Plates were washed and horseradish peroxidase (HRP)-conjugated goat anti-monkey IgG (Alpha Diagnostics) or anti-monkey IgM (Alpha Diagnostics) diluted 1:10000 was added and incubated 1 hour at RT. Plates were washed and the enzyme reaction was initiated using KPL 3,3′,5,5′-tetramethylbenzidene (TMB) substrate (SeraCare) and stopped with 2 M H_2_SO_4_. Antibody concentrations were determined using a standard curve derived from purified rhesus IgG or monkey IgM whole molecule obtained from the NHP Reagent Resource as previously described^65^. The fold change in antibody levels was calculated based on antibody present in B cell only supernatants.

### Quantification of gp120-specific Ab in rectal secretions

Rectal swabs were stored at −80 °C and were thawed before use. All samples were tested for blood contamination using Chemstrip 5OB (Roche, Indianapolis, IN). Samples containing >50 erythrocytes/ml were not analyzed. Ninety-six–well ELISA plates were coated with 50 µl of 1 µg/ml gp120_CG7V_ or gp120_M766_ in carbonate-bicarbonate buffer (pH 9.6) overnight at 4 °C. Rectal secretions were serially diluted in D-PBS containing 1% BSA and 0.05% Tween20. 50 µl of the serial two-fold dilutions were incubated on the plates for 1 h at 37 °C. The plates were then washed 4 times and 50uL of HRP-conjugated goat anti-monkey IgG or IgA (Alpha Diagnostic) at 1:10000 dilution in 1% BSA solution were added. Plates were incubated at room temperature for 1 h, washed 4 times, and TMB substrate (KPL) was used in sequential steps, followed by reading the OD at 450 nm. For total IgG and IgA antibody, standards were obtained from the Nonhuman Primate Reagent Resource and goat anti-monkey HRP conjugates were used as detection antibodies at a 1:10000 dilution. Env-specific IgG and IgA, derived from purified serum IgG and IgA obtained from SIV_mac251_-infected macaques and quantified as described^66^, was used to generate a standard curve for Env-specific IgG and Env-specific IgA. Mucosal antibodies were reported as ng Env-specific IgG or IgA per ug total IgG or IgA.

### Rectal SIV-specific memory B cell detection

Rectal cells were obtained 21 days post-immunization to detect SIV-specific memory B cell responses. For antigen-specific memory B cell staining, the cells were blocked with anti-CD4 blocking antibody for 20 minutes at 4°C, followed by washing and incubating with biotinylated SIV_CG7V_ gp120, or biotinylated SIV_M766_ gp120 and then stained with APC streptavidin. The cells were stained with Live/Dead aqua dye followed by surface staining with Alexa Fluor 700 anti-CD3 (SP34-2), BV650 anti-CD20 (2H7), BV605 anti-CD21 (B-ly4) (BD Biosciences), PE-Cy5 anti-CD19 (J3-119) (Beckman Coulter), and PE-TxRed anti-IgD (AT-1) (StemCell). Acquisition and analysis followed the same protocol as for MAIT cells.

### Statistical analysis

The Mann–Whitney U test was used for comparisons between different groups of animals, and the Wilcoxon signed rank test was used for paired differences within the same group of animals. Correlations were assessed using the Spearman rank correlation test. Analyses were performed using GraphPad Prism (GraphPad Software).

## Supplementary information

Supplementary Information.

## Data Availability

The datasets generated during and/or analysed during the current study are available from the corresponding author on reasonable request.
