# Supplementary material for: Mucosal-associated invariant T (MAIT) cells provide B-cell help in vaccinated and subsequently SIV-infected Rhesus Macaques
Source: Sci Rep. 2020 Jun 22;10:10060. doi: 10.1038/s41598-020-66964-0 (PMC7308357; doi:10.1038/s41598-020-66964-0)
Supplement: Supplementary file 1 — Supplementary Information. [file 41598_2020_66964_MOESM1_ESM.pdf]

Mucosal-associated invariant T (MAIT) cells provide B-cell help in vaccinated and subsequently SIV-infected Rhesus Macaques

Mohammad Arif Rahman[1], Eun-Ju Ko[1#], Farzana Bhuyan[2], Gospel Enyindah-Asonye[1], Ruth Hunegnaw[1], Sabrina Helmold Hait[1], Christopher Hogge[1], David J. Venzon[3], Tanya Hoang[1] and Marjorie Robert-Guroff[1*]

[1]Vaccine Branch, Center for Cancer Research, National Cancer Institute, National Institutes of Health, Bethesda, MD

[2]Laboratory of Clinical Immunology & Microbiology, National Institute of Allergy and Infectious Diseases, National Institutes of Health, Bethesda, MD

[3]Biostatistics and Data Management Section, National Cancer Institute, National Institutes of Health, Bethesda, MD 20892.

[#]Present address: College of Veterinary Medicine and Interdisciplinary Graduate Program in Advanced Convergence Technology & Science, Jeju National University, Jeju, 63243, Korea

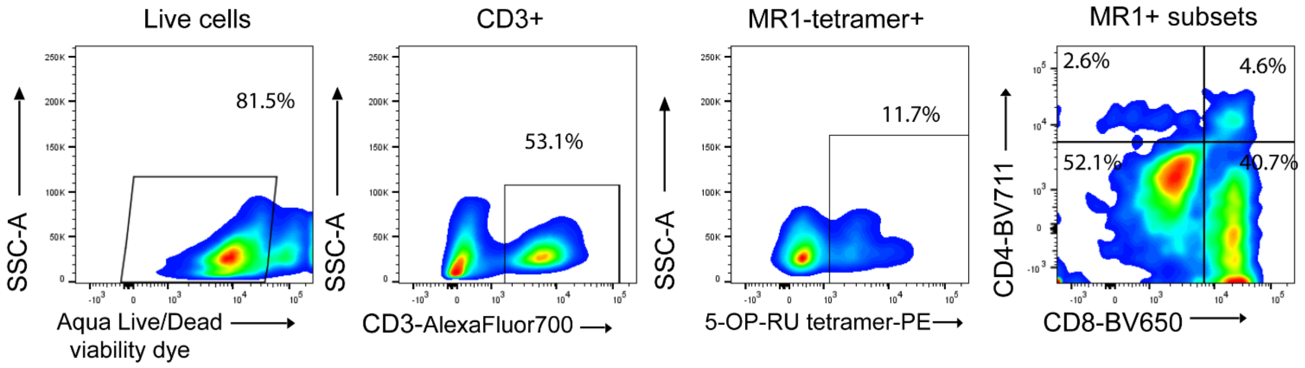

Supplementary Figure S1. Gating strategy for CD8+CD4-, DP (CD8+CD4+), CD8-CD4+ and DN (CD8-CD4-) MAIT cell subgroups in blood and BAL. The figure illustrates gating of MAIT cells in BAL fluid.

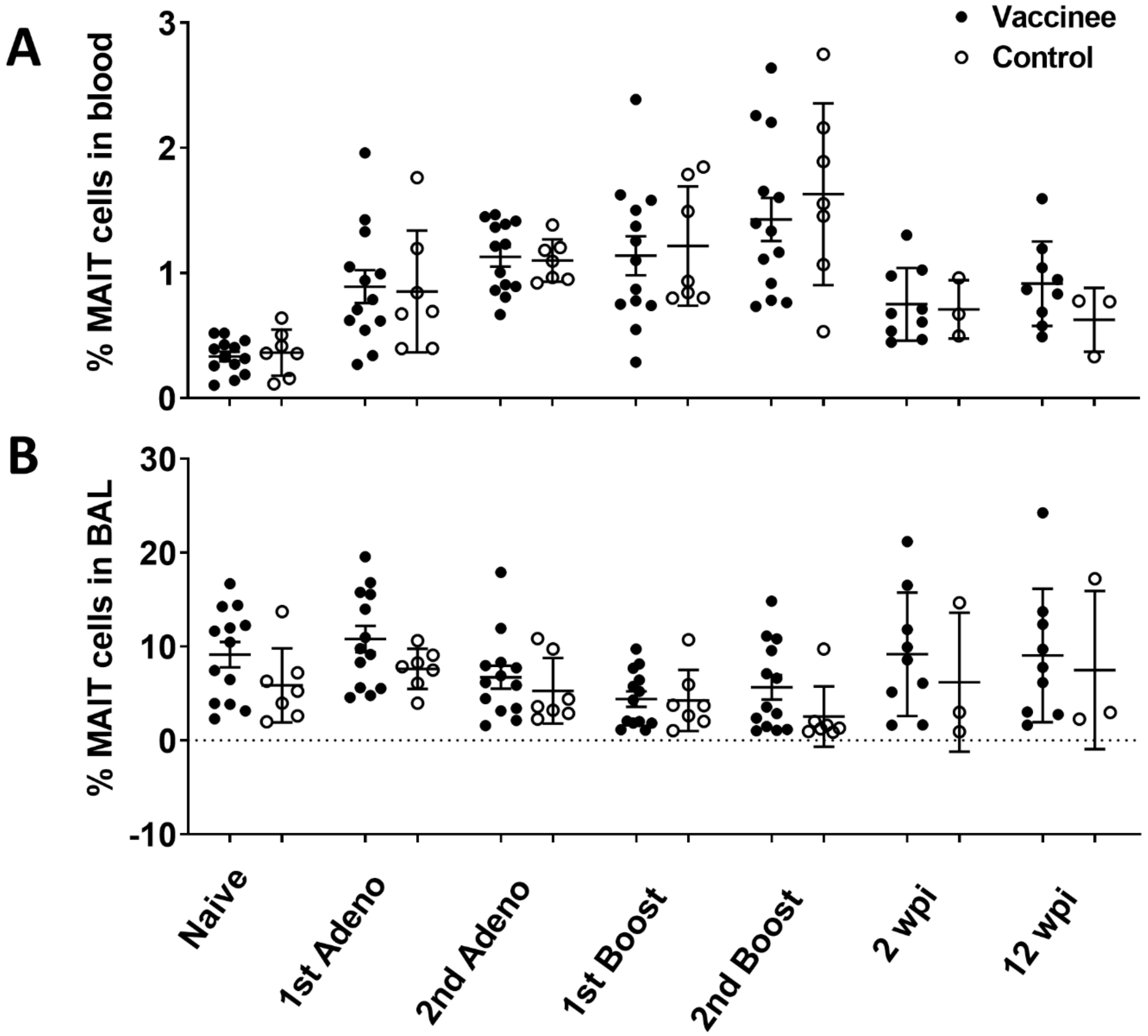

Supplementary Figure S2. Comparison of MAIT cell frequencies between vaccinated and control animal groups in (A) blood and (B) BAL fluid upon vaccination and subsequent SIV infection in rhesus macaques. Naïve and during vaccination, vaccine group N=13 and control group N=7; after infection, vaccine group N=9 and control group N=3. No significant differences were observed between vaccine and control groups at any timepoint.

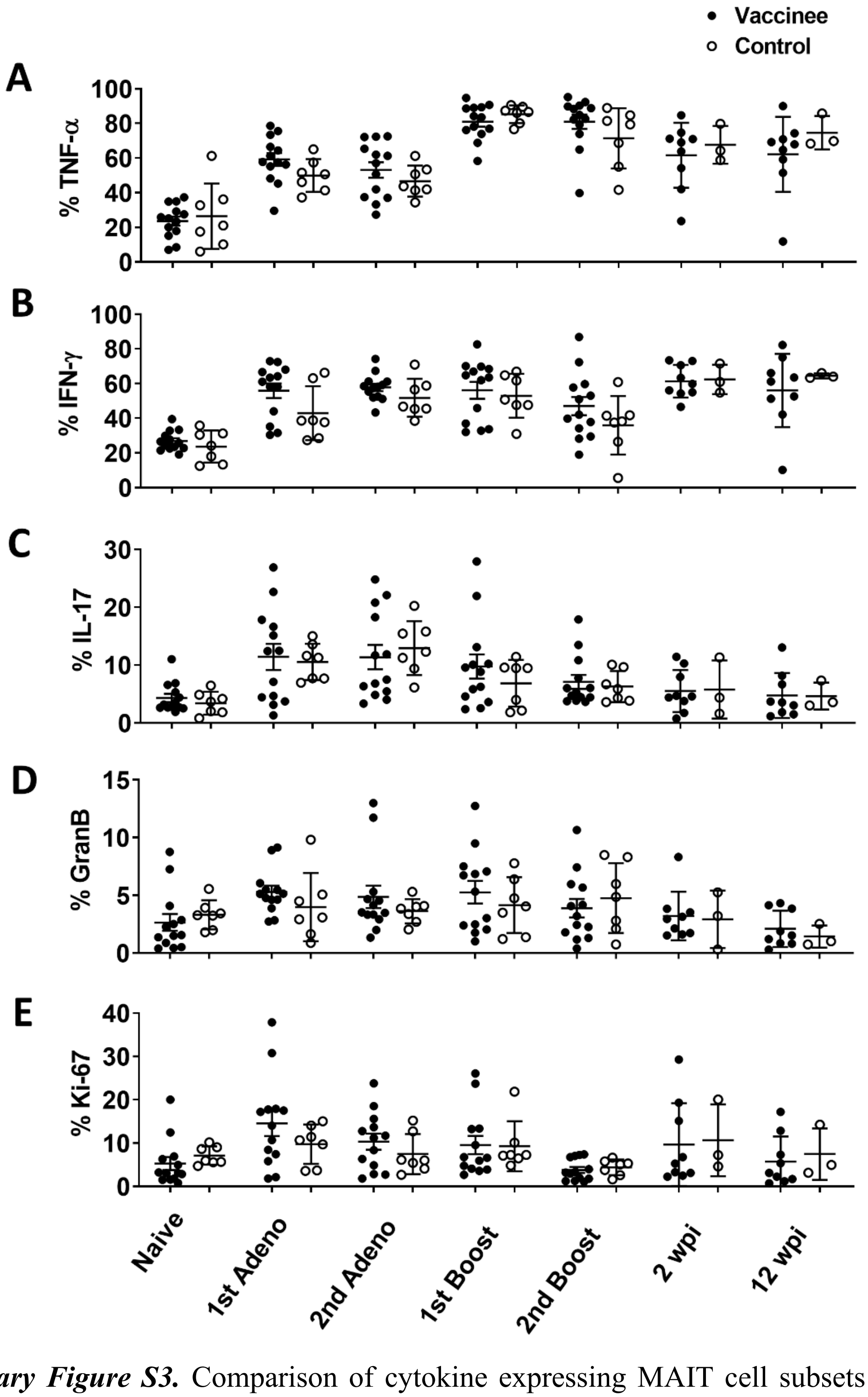

Supplementary Figure S3. Comparison of cytokine expressing MAIT cell subsets in blood of vaccinated and control animals. Frequency of (A) TNF-α^+, (B) IFN-γ^+, (C) IL-17^+, (D) Granzyme B^+ and (E) Ki-67-expressing MAIT cells were comparable between vaccine and control groups at each timepoint. Naïve and during vaccination, vaccine group N=13 and control group N=7; after infection, vaccine group N=9 and control group N=3.

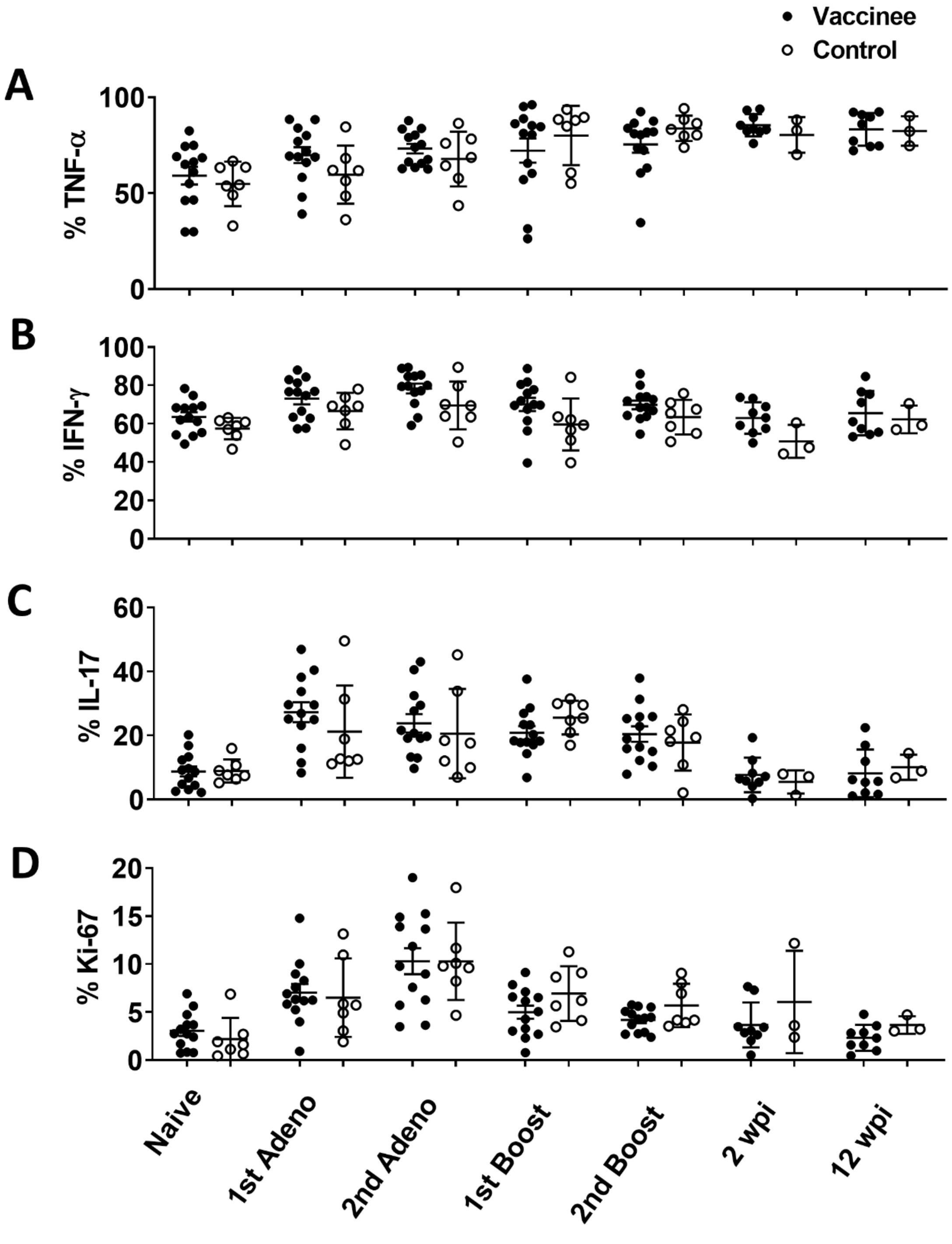

Supplementary Figure S4. Comparison of cytokine expressing MAIT cell subsets in BAL of vaccinated and control animals. Frequency of (A) TNF-α$^+$, (B) IFN-γ$^+$, (C) IL-17$^+$, and (D) Ki-67-expressing MAIT cells were comparable between vaccine and control groups at each timepoint. Naïve and during vaccination, vaccine group N=13 and control group N=7; after infection, vaccine group N=9 and control group N=3.

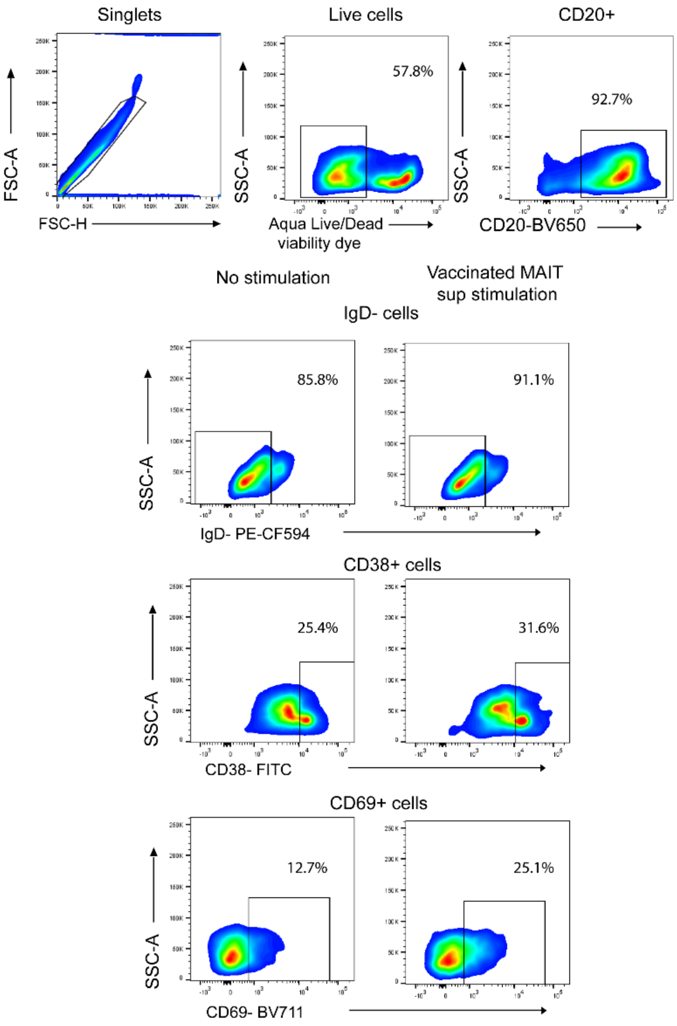

Supplementary Figure S5. Gating strategy of IgD negative, $CD38^{+}$ and $CD69^{+}$ B cells.

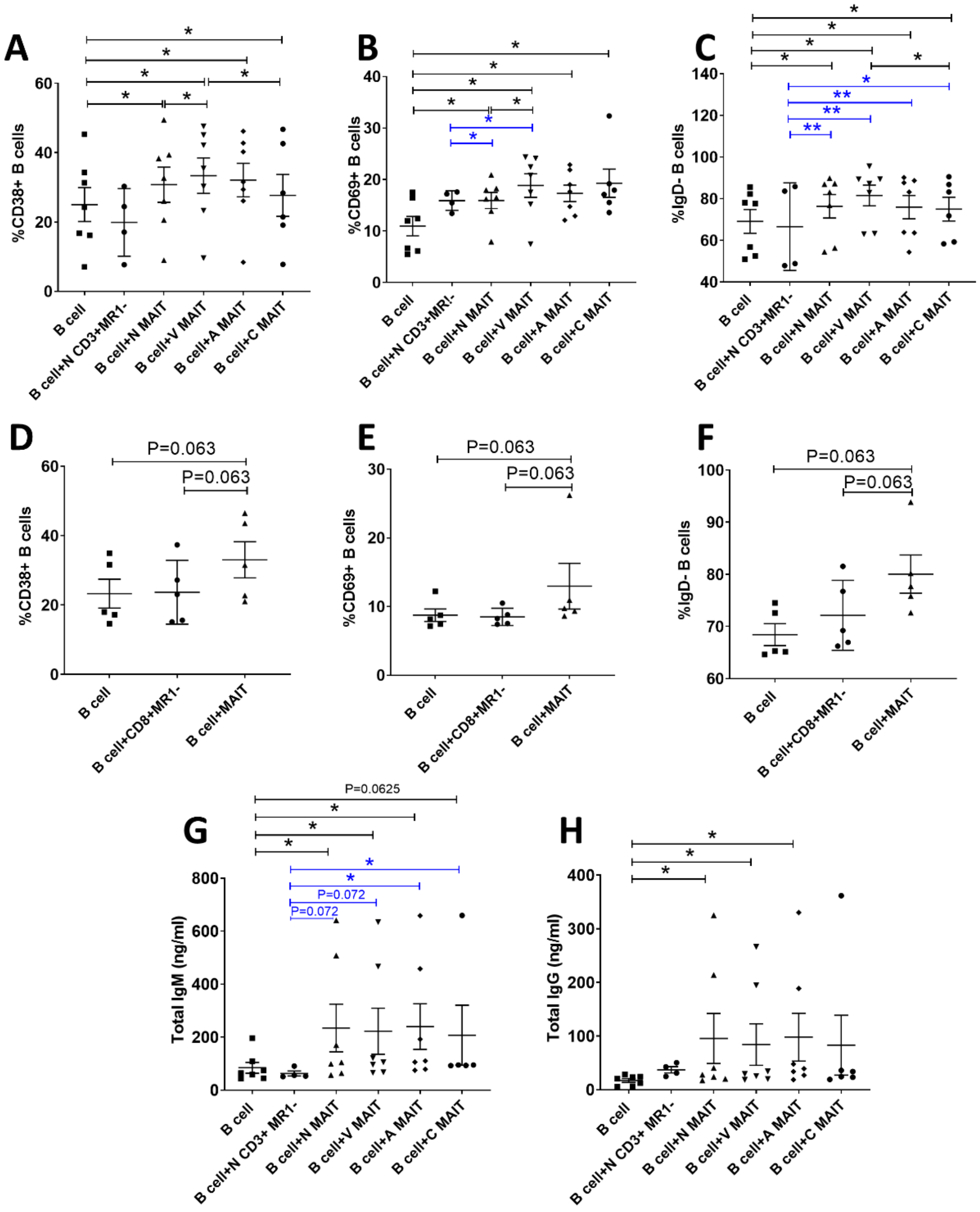

Supplementary Figure S6. Influence of MAIT cells on B cell activation and antibody secretion. Following stimulation with enriched (A-C) or sorted (D-E) MAIT cell supernatants, the frequencies of naïve B cells expressing (A, D) CD38, (B, E) CD69 and (C, F) IgD exhibited changes. B cells stimulated with supernatants of enriched MAIT cells from naïve, vaccinated, acute and chronically infected macaques were compared with B cells cultured alone or with B cells cultured in the presence of enriched naïve $CD3^{+}MR1^{-}$ cell supernatants (A-C). B cells cultured with sorted MAIT cell supernatants were compared with B cells cultured alone or with B cells cultured with sorted $CD8^{+}MR1^{-}$ cell supernatants (D-F). B cells stimulated with enriched MAIT cell supernatant secrete (G) IgM and (H) IgG. Data of (A-H) were analyzed by the Wilcoxon paired test or Mann–Whitney U test. Horizontal and vertical bars denote mean and SEM. *$p < 0.05$.
